# Bio-Based Nanoparticles as a Carrier of β-Carotene: Production, Characterisation and In Vitro Gastrointestinal Digestion

**DOI:** 10.3390/molecules25194497

**Published:** 2020-09-30

**Authors:** Beatriz S. Afonso, Ana G. Azevedo, Catarina Gonçalves, Isabel R. Amado, Eugénio C. Ferreira, Lorenzo M. Pastrana, Miguel A. Cerqueira

**Affiliations:** 1 CEB—Centre of Biological Engineering, University of Minho, 4710-057 Braga, Portugal; a75621@alunos.uminho.pt (B.S.A.); ecferreira@deb.uminho.pt (E.C.F.); 2 INL—International Iberian Nanotechnology Laboratory, 4715-330 Braga, Portugal; ana.azevedo@inl.int (A.G.A.); catarina.goncalves@inl.int (C.G.); isabel.rodriguez@inl.int (I.R.A.); lorenzo.pastrana@inl.int (L.M.P.)

**Keywords:** encapsulation, nanotechnology, bioactive compounds, zein, ethylcellulose, biopolymers

## Abstract

β-carotene loaded bio-based nanoparticles (NPs) were produced by the solvent-displacement method using two polymers: zein and ethylcellulose. The production of NPs was optimised through an experimental design and characterised in terms of average size and polydispersity index. The processing conditions that allowed to obtain NPs (<100 nm) were used for β-carotene encapsulation. Then β-carotene loaded NPs were characterised in terms of zeta potential and encapsulation efficiency. Transmission electron microscopy, Fourier transform infrared spectroscopy and X-ray diffraction analysis were performed for further morphological and chemical characterisation. In the end, a static in vitro digestion following the INFOGEST protocol was performed and the bioaccessibility of β-carotene encapsulated in both NPs was determined. Results show that the best conditions for a size-controlled production with a narrow size distribution are lower polymer concentrations and higher antisolvent concentrations. The encapsulation of β-carotene in ethylcellulose NPs resulted in nanoparticles with a mean average size of 60 ± 9 nm and encapsulation efficiency of 74 ± 2%. β-carotene loaded zein-based NPs resulted in a mean size of 83 ± 8 nm and encapsulation efficiency of 93 ± 4%. Results obtained from the in vitro digestion showed that β-carotene bioaccessibility when encapsulated in zein NPs is 37 ± 1%, which is higher than the value of 8.3 ± 0.1% obtained for the ethylcellulose NPs.

## 1. Introduction

Nanoencapsulation is presented as one of the ways to improve the bioaccessibility of several lipophilic bioactive compounds. This improvement is a consequence of an increased solubility resulting from the encapsulation systems that are easily dispersed in aqueous solutions and due to its protection effect when submitted to harsh conditions (food processing or gastric phase during digestion) and the better micellarisation during the intestine phase [1]. Several works showed the possibility of producing delivery systems able to encapsulate bioactive compounds using food grade and bio-based materials. The selection of a suitable method for the preparation of polymeric nanoparticles is made based on the type of polymer, size requirement, the simplicity of the procedure, area of application and consequently the safety of the materials used [2,3].

The solvent displacement method, also known as nanoprecipitation, was described firstly by Fessi et al. [4] and consists on a method for the development of nanoparticles in a reproducible, easy and scalable way. Since there is an increasing interest to employ processes that are environmentally sustainable, safe and energy-saving, the solvent displacement method has been widely used. This method enables the production of nanospheres as well as nanocapsules in one cost-efficient process, with a high yield encapsulation of hydrophobic compounds [5]. The nanoprecipitation method requires three basic ingredients to be performed: the polymer, the solvent and the antisolvent (also known as non-solvent) of the polymer. In the process of selecting of a suitable solvent it should be insured that its complete miscibility with the antisolvent and able to solubilise the bioactive compound. Moreover, the antisolvent must have a low boiling point allowing its removal by evaporation [6]. The polymer, the bioactive compound to be encapsulated and the solvent constitute the organic phase. The antisolvent of the polymer constitutes the aqueous phase which is usually water. Other materials could be added to this phase, such as hydrophilic surfactants to avoid particle aggregation and coating materials [7]. Several operating conditions influence the characteristics of the nanoparticles such as, the ratio of organic phase to the aqueous phase, stirring rate, the injection rate of the organic phase, which phase is poured and polymer concentration [2]. Several polymers have been tested for the production of particles and capsules using the nanoprecipitation method, as reported elsewhere [8]. Two of these polymers are ethylcellulose and zein.

Ethylcellulose is a semi-synthetic material derived from cellulose, which is biocompatible, non-toxic, water-insoluble, biodegradable and with wall-forming properties [9,10]. Moreover, it is a tasteless, white to light tan-coloured powder presenting a wide range of applications in the food, cosmetic and pharmaceutical fields [11,12]. Ethylcellulose is soluble in a wide variety of solvents such as aromatic hydrocarbons, alcohols, ketones and chlorinated solvents. It is also soluble in ethanol and methanol yielding solutions with lower viscosity [13]. Moreover, because ethylcellulose is insoluble in water it is widely used for the controlled release of hydrophobic bioactive compounds [14,15]. Ethylcellulose is approved as a food additive in Europe (E462) [16], considered “Generally Recognised as Safe” GRAS and approved by the FDA (The United States Food and Drug Administration) as safe for human consumption [17].

Zein belongs to the group of corn proteins named prolamins [18] and its constituted by a mixture of four different classes grouped by solubility and sequence similarity, namely α-, β-, γ- and δ -zeins. α-zein is normally the most abundant fraction [19]. Zein is an amphiphilic biopolymer since it has both hydrophobic and hydrophilic attributes. The hydrophobic aminoacid presented are leucine, proline and alanine and the hydrophilic counterpart is represented by glutamine [20]. However, zein almost completely lacks lysine and tryptophan amino acids which is why it has poor nutritional quality [21]. The combination of zein’s different amino acids is responsible for its particular solubility. Zein is insoluble in pure water and pure ethanol, but is soluble in aqueous-ethanol solutions at specific concentrations making it a material of interest for the controlled release of drugs [22]. Zein is a renewable, biodegradable and biocompatible [20] powder with a yellow colour due to the carotenoids present in the corn [23]. In addition, it is thermally stable up to 280 °C, shows excellent oxygen barrier properties [20] and can withstand gastric pH [24]. Zein has been regarded with GRAS status by the Food & Drug Administration [25].

β-carotene is currently used as a food additive with the European denomination E160 [16] acting as a colouring agent, antioxidant and vitamin A precursor [26]. β-carotene does not have recommended dietary allowance, however, the recommendation for vitamin A for women and men is 800 and 1000 μg of retinol (or retinol equivalents), respectively [27]. Assuming that all β-carotene consumed is converted to retinol, the dietary allowance would be approximately 4.8 and 6.0 mg for women and men, respectively [28]. One of the problems of β-carotene is its instability to harsh conditions such as processing temperatures and gastric conditions. Therefore, the encapsulation was presented as one of the ways to protect this kind of compounds during the Human digestion, resulting in an increased bioaccessibility and bioavailability [1,3,29]. The possibility of encapsulating β-carotene in nano delivery systems has been highly explored, however only few works reach the evaluation of its bioaccessibility in gastrointestinal conditions [30,31,32]. To the best of the authors’ knowledge, the evaluation of bioaccessibility of β-carotene-loaded in zein and ethylcellulose nanoparticles and its comparison has never been performed.

Therefore, in this work nanoparticles based on ethylcellulose and zein were produced, characterised and used to encapsulate β-carotene. Their size distribution, polydispersity and zeta potential were determined and TEM, FTIR and XRD were used to confirm their morphology and assess chemical characteristics. In the end, an in vitro gastrointestinal model (static) was used to evaluate the bioaccessibility of β-carotene encapsulated within both nanosystems, after digestion.

## 2. Results and Discussion

### 2.1. Optimisation of Nanoparticles (NPs) Production

**Figure 1:**
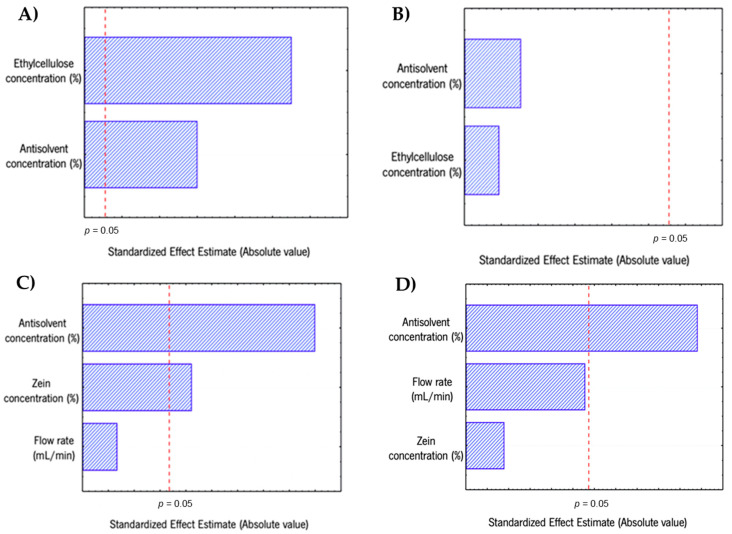
Pareto chart of standardised effects for (**A**) size of ethylcellulose nanoparticles, (**B**) PDI of ethylcellulose nanoparticles, (**C**) size of zein nanoparticles and (**D**) PDI of zein nanoparticles.

Aiming to obtain NPs with low size and polydispersity index (PDI) values, different processing parameters were evaluated for the production of ethylcellulose and zein NPs. For ethylcellulose NPs production, the polymer and antisolvent concentrations were considered the independent variables as presented in detail in the Section 3.5. Figure 1A,B show the effect of varying the antisolvent and polymer concentration on size and PDI values, respectively. Polymer concentration varied between 0.1 and 0.4% (*m*/*v*) and antisolvent concentration varied between 60, 70 and 80% (*v*/*v*). Results show that the parameters that influence the size of the ethylcellulose NPs are both ethylcellulose concentration and antisolvent concentration, that present statistical significance (*p* < 0.05) as presented in Figure 1A and in detail in Table S1. This phenomenon can be explained by the increase of the polymer concentration that results in a higher viscosity of the organic phase that hampers the solvent diffusion into the water, lowering the nucleation rate and producing bigger aggregates [33]. Moreover, increasing the antisolvent concentration will induce a faster solvent diffusion into the water and smaller particles are formed [7]. Since the aim was to obtain low sizes with a narrow size distribution, both parameters were considered and were combined. Therefore, lower ethylcellulose concentrations (0.1%) and higher antisolvent concentrations (80%) were used. These conditions allow the production of ethylcellulose NPs with 69 ± 2 nm and a PDI of 0.18 ± 0.04. The average size is lower than the ones presented elsewhere, for ethylcellulose NPs produced by nanoprecipitation (using acetone as solvent) [34] and high-pressure emulsification solvent evaporation [35], which obtained sizes of 163 nm and 150 nm, respectively; the same size range is obtained for NPs produced by electrospray [36] and nanoprecipitation (using ethanol as solvent) [37].

During the optimisation of the zein NPs production, the polymer and antisolvent concentrations and the flow rate were considered the independent variables as presented in detail in the Section 3.5. Polymer concentration varied between 0.4 and 0.8% (*m/v*), antisolvent concentration varied between 80 and 90% (*v/v*) and flow rate varied between 0.3 and 0.7 mL/min. Figure 1C,D present the effect of varying those variables on average size and PDI, respectively. It is shown that the parameters influencing (*p* < 0.05) the NPs size are the antisolvent and zein concentrations; this behaviour was also observed for the production of ethylcellulose NPs. The results show that higher concentrations of antisolvent lead to a significant decrease on the size, e.g., using 0.7 mL/min flow rate and 0.4% (*m/v*) zein concentration, the NPs average size goes from approximately 140 to 90 nm, when the antisolvent concentration goes from 80% to 90%. Lower concentrations of zein also revealed smaller NPs sizes (Table S2). These results are similar to the ones presented for ethylcellulose NPs and can be explained by the same phenomenon, where lower polymer concentration and higher amounts of antisolvent reduces the viscosity of the suspension inducing fast miscibility of phases and smaller particles are formed. Also, when less zein and more water is used, it results in less accessible zein polymer, thus decreasing the agglomeration of particles as well as their size. Similar results were presented elsewhere, where an increase of zein concentration led to higher particle sizes [19]. Results obtained in the present study showed that the conditions and methodology used result in NPs with lower sizes than others obtained by similar methods and different methods such as electrospray [38,39]. Although similar low sizes were obtained by Cheng et al. [40] with particle size values of around 72 nm using the nanoprecipitation method with simultaneous high shear (the authors used a benchtop homogenizer (Ultra Turrax T25 Basic, Long Island, NY, USA).

Regarding PDI, none of the variables tested for ethylcellulose had a significant influence over the PDI (*p* > 0.05) (Figure 1B). In the case of zein NPs it was observed that the PDI was influenced by the antisolvent concentration (*p* < 0.05). Results show that a higher amount of antisolvent resulted in higher PDI, varying from 0.29 to 0.20, at 0.7 mL/min flow rate and 0.4% zein concentration. PDI measures the homogeneity of the NPs dispersion, PDI values greater than 0.5 can represent aggregation of particles [41] but for the tested conditions all measurements stayed below this value.

Based on results obtained and considering that the aim was to obtain low particle sizes and PDI, the production conditions for the further tests were 0.1% (*m/v*) of polymer and 80% (*v/v*) of antisolvent for ethylcellulose NPs while in the case of zein NPs was used 0.4% (*m/v*) of polymer, 90% (*v/v*) of antisolvent and a flow rate of 0.7 mL/min.

### 2.2. Encapsulation of β-Carotene

β-carotene was dissolved in the organic phase and loaded in the ethylcellulose and zein NPs during the production process. In a first stage, different concentrations of β-carotene were tested (ranged between 4 and 0.4 μg/mL for EC and 5 and 10 μg/mL for zein), aiming higher loads and encapsulation efficiency (EE), while keeping similar sizes and PDI values than the unloaded NPs. For ethylcellulose nanoparticles, it was observed that a β-carotene concentration of 0.4 μg/mL resulted in an EE of 75 ± 18% and for zein NPs 5 μg/mL resulted in 89 ± 12% of EE. Bourbon et al. [42] and Souza et al. [43] showed that the EE curves typically reach a peak at a certain (optimal) bioactive concentration, and then drop into much lower values. However, here, in both types of NPs high concentrations of β-carotene results in similar (*p* > 0.05) EE values. For ethylcellulose NPs the use of 4 μg/mL of β-carotene led to an EE of 74 ± 2% while for zein NPs the use of 10 μg/mL of β-carotene resulted in a EE of 93 ± 4%. Similar results were obtained by Wang et al. [44] for zein nanoparticles, where a maximum EE (approx. 50%) was obtained for a 1:5 mass ratio between β-carotene and zein. Wu et al. [39] encapsulated thymol and carvacrol in zein nanoparticles and obtained values ranging from ≈50 to ≈90%.

Regarding the physical parameters, the use of high concentrations of β-carotene results in aggregates and high PDI values for both systems. The size distribution, PDI and zeta potential of the unloaded and loaded nanoparticles were determined and presented in Table 1. Loaded NPs were prepared using optimal conditions presented in the previous section.

Ethylcellulose NPs showed similar size and PDI values to the respective loaded NPs. However, the PDI increased after NPs loading, which may be due to the surface area variation that can occur as a result of β-carotene addition.

Zein nanoparticles demonstrated similar sizes and PDI for unloaded and loaded nanoparticles. Since the nanoparticles’ size were maintained and all PDI values were lower than 0.5, the loaded nanoparticles were used for further studies. Similar results were presented by Cheng et al. [40] for zein nanoparticles showing that size and PDI were maintained after loading with lutein (another carotenoid).

In order to evaluate the NPs stability in aqueous dispersion, the zeta potential was determined. It has been reported that the zeta potential can be an indicator of how stable a dispersion of nanoparticles is, i.e., higher absolute values indicate higher repulsion between particles, meaning that the suspension is stable. In general, zeta potential values outside the range +30 mV to −30 mV are associated with high stability [45]. The zeta potential values obtained for unloaded and loaded NPs are different (*p* < 0.05). The incorporation of β-carotene leads to an increase (considering the absolute value) of the values in both cases, from −61 mV to −93 mV for ethylcellulose NPs and from +64 mV to +70.5 mV for zein NPs. These changes can be related to the presence of β-carotene at the surface of NPs. This can influence the stability of the NPs, but in this case, the loaded-NPs maintain higher values of zeta potential and therefore no changes on the stability are foreseen.

**Figure 2:**
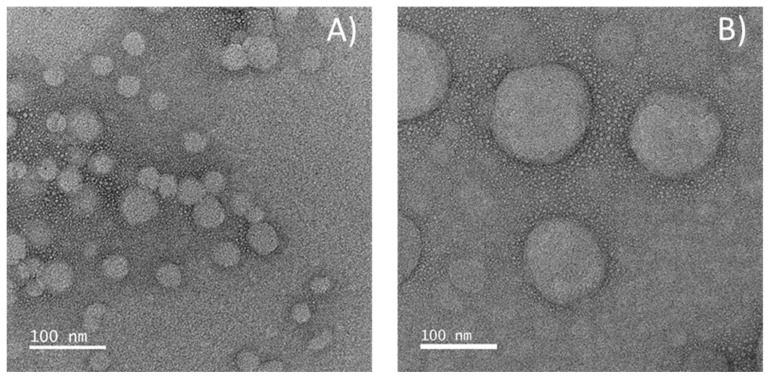
TEM images of (**A**) loaded ethylcellulose nanoparticles and (**B**) loaded zein nanoparticles. Magnification of 100,000×.

Figure 2 confirms the spherical shape of the nanoparticles. The sizes are consistent with the results obtained by DLS; it can be seen a broad range of particles sizes in agreement with the polydispersity values obtained by DLS, however the drying process required for microscopy observation could also change the particles size. It is also confirmed by the images the lower size of ethylcellulose NPs when compared with zein NPs, as determined by DLS. The morphology observed is in line with the TEM images presented elsewhere for zein NPs [19] and ethylcellulose NPs [46].

### 2.3. Fourier Transform Infrared Spectroscopy and X-ray Diffraction

**Figure 3:**
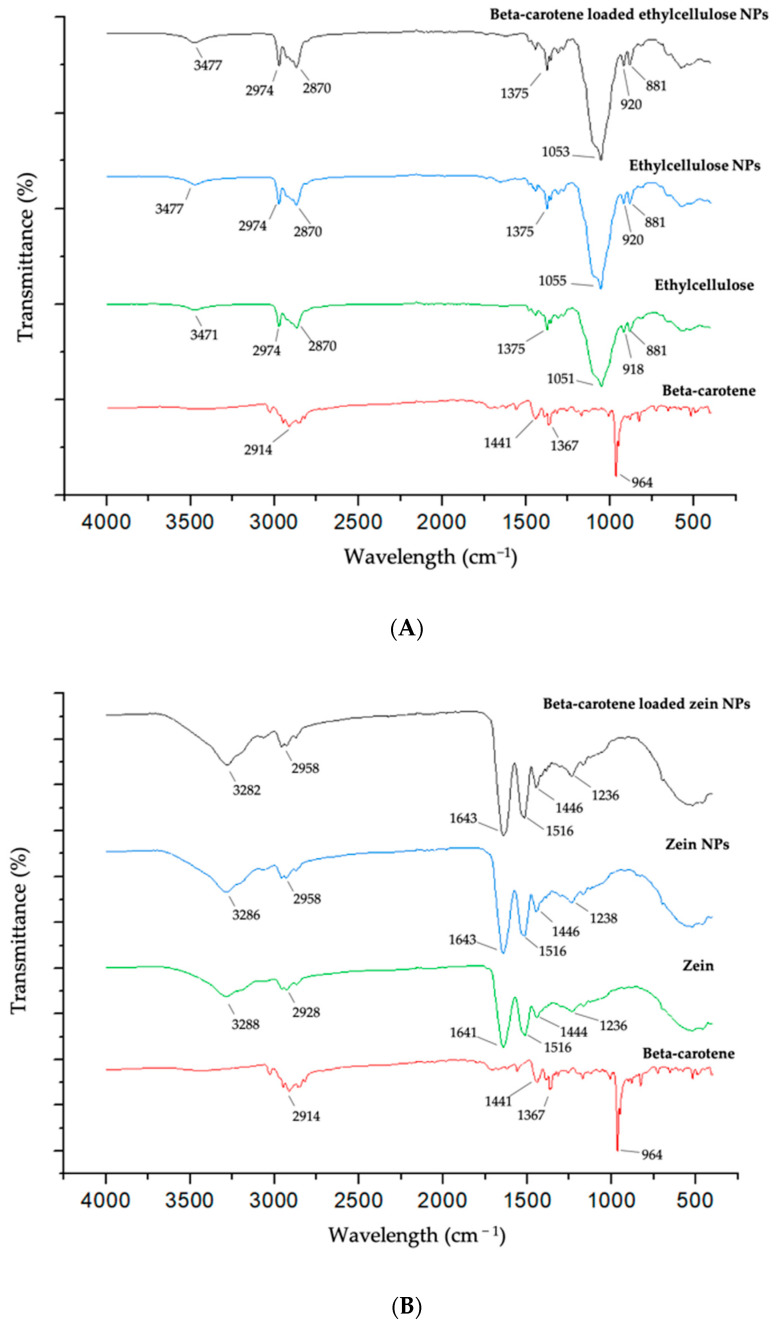
FTIR spectra of (**A**) β-carotene, ethylcellulose, ethylcellulose nanoparticles (NPs) and β-carotene loaded ethylcellulose NPs and (**B**) β-carotene (BC), zein, zein NPs and β-carotene loaded zein NPs.

The β-carotene spectra show a clear peak at 964 cm^−1^, which is the trans conjugated alkene CH out of plane deformation mode [47]. The absorption characteristic peaks of ethylcellulose (Figure 3A) at 1051 cm^−1^ corresponds to C–O–C stretching and the wavelengths 2870 cm^−1^ and 2974 cm^−1^ corresponded to C–H. The band at 3471 cm^−1^ is due to the hydroxyl (O–H) stretching vibrations [48]. From pure ethylcellulose to the NP form there was only a peak shift from 3471 cm^−1^ to 3477 cm^−1^. These results reveal no significant change in the structure of ethylcellulose when nanoparticles are produced. Even as loaded β-carotene NPs, no significant β-carotene peaks were found. This may be due to the small amount of β-carotene in the sample when compared to ethylcellulose, since the intensity of the peaks of FTIR spectra are expected to be proportional to the amount of the compound in the tested sample. Moreover, the state of the samples and the way they are contacting the crystal can influence this result.

For pure zein, the characteristic bands of proteins can be seen in Figure 3B. Protein bands that most stand out are amide I or C=O, usually in the range 1600–1700 cm^−1^, stretching at 1641 cm^−1^ and amide II or N–H, usually present at 1500–1530 cm^−1^, bending and stretching at 1516 cm^−1^. Amide A or N–H is stretching at 3288 cm^−1^; amide B or asymmetric stretching vibration of =C–H at 2928 cm^−1^; and amide III or C–N stretching at 1236 cm^−1^. Similar values can be seen in the encapsulation structures containing zein [30,47].

The shift from 2928 to 2958 cm^−1^ when the material was in the form of NP can implies that the formation of nanoparticles or the production process can impact the protein structure, since represents a change in the amide B or asymmetric stretching vibration of =C–H. In the loaded β-carotene NPs, there was no noticeable difference in the amide bands, implying that the β-carotene incorporated in the polymer may not show an obvious effect on the proteins structure when compared with from the unloaded NPs or that the concentration of β-carotene used in the NPs hampered the unequivocal identification of β-carotene in the FTIR spectra of encapsulated particles [30].

**Figure 4:**
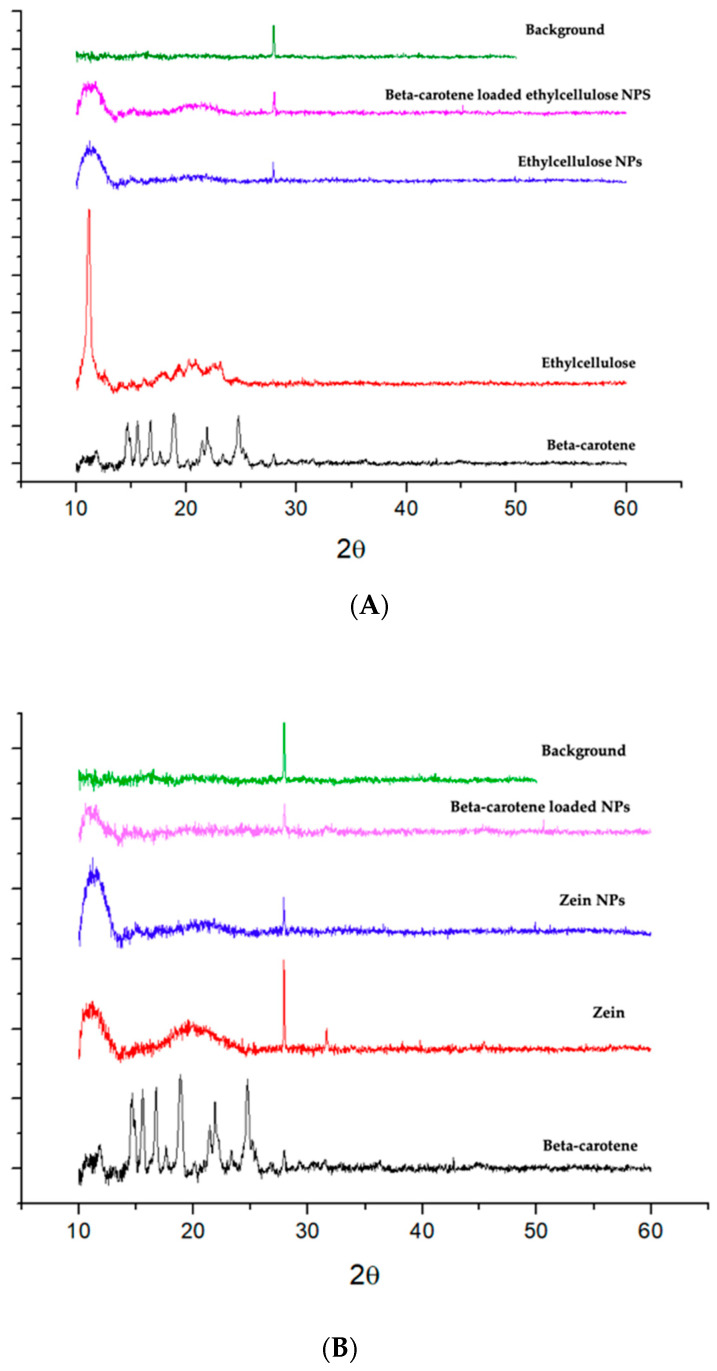
X-ray diffraction patterns of (**A**) β-carotene, ethylcellulose, ethylcellulose NPs and β-carotene loaded ethylcellulose NPs and (**B**) β-carotene, zein, zein NPs and β-carotene loaded zein NPs.

The X-ray spectrum (Figure 4) of the pure β-carotene showed that the compound is a crystalline material. However, the β-carotene peaks were not visible in the loaded β-carotene nanoparticles, either for ethylcellulose or zein nanoparticles suggesting that β-carotene is amorphous in both nanosystems. Changes in β-carotene crystallinity may be due to its precipitation without crystallisation when in contact with the antisolvent or during solvent evaporation [47]. Pure zein spectrum did not show sharp peaks, instead, it shows two humps, indicating the amorphous structure of the protein [38]. Zein nanoparticles and loaded nanoparticles show similar amorphous spectra. Pure ethylcellulose spectrum revealed sharp peaks, which confirmed the presence of crystalline structure [49]. All NPs showed to be amorphous, however, ethylcellulose characteristic peak showed an observable intensity that can be also seen in ethylcellulose NPs and loaded ethylcellulose NPs. Results reported by Tao et al. [50] revealed that nanoparticles produced by nanoprecipitation were usually amorphous and less stable during storage compared to their crystalline counterpart. Since amorphous compounds easily recrystallise in water, and therefore increase aggregation, a desirable solution is to freeze-dry the samples for an improved stability. The use of dried amorphous loaded-NPs can help their re-suspension and therefore their further use in aqueous media. However, Yi et al. [47] reported that an amorphous state could be desirable since the bioavailability of amorphous β-carotene is expected to be greater than crystalline β-carotene.

### 2.4. In Vitro Gastrointestinal Digestion

One of the aims of the encapsulation of bioactive compounds is their protection against harsh conditions during processing or under digestive conditions. The gastrointestinal conditions can change the chemical structure of bioactive compounds, but also increase their solubility and therefore improve their bioaccessability. Therefore, the determination of the bioaccessibility of an encapsulated compound is one of the ways to assess if the encapsulation system is effective on the protection of the bioactive compound that must reach the intestine in its active form, being ready to be absorbed.

The INFOGEST protocol [51] was used to assess the bioaccessibility of β-carotene loaded in the two types of nanoparticles. The protocol aims to quantify the amount of β-carotene in the micellar phase at the end of the digestion. β-carotene is highly hydrophobic and needs to resist the gastric conditions and be incorporated within the mixed micelles during the digestion process to reach the target site. Efficient micellisation of β-carotene during digestion is crucial for its bioaccessibility [52].

The oral phase is meant to simulate the hydration and lubrication of food in the mouth in order to obtain an adequate moisture to facilitate the mixing in the gastric phase. Therefore, this phase does not influence the output of the digestion of these nanoparticles. In the gastric phase, the bolus is diluted by the simulated gastric fluid, pepsin is added, and the pH is adjusted to 3.0. In the intestinal phase, the chyme from the previous phase is mixed with bile and pancreatic juice. Bile is crucial to emulsify fat and form mixed micelles that transport β-carotene through the intestinal epithelium [51]. Table 2 shows the bioaccessibility of β-carotene at the gastric and intestinal phases for each type of NP.

Ethylcellulose NPs presented low bioaccessibility of β-carotene. Indeed, ethylcellulose is insoluble in water at any physiological pH, and there are no digestive enzymes able to metabolize ethylcellulose. However, it can swell in the presence of gastric juice making it permeable for water and allowing β-carotene diffusion and release [53]. That should be the reason for the low amount of β-carotene obtained in the gastric (2.7%) and intestinal (8.3%) phases, respectively (Table 2). It is also important to mention that the detection of β-carotene in the gastric phase might result from the presence of some non-encapsulated β-carotene in the system, and not as a consequence of polymer digestion. However, the amount of β-carotene in the intestinal phase (bioaccessibility) increased significantly, which is in agreement with the expected behaviour for these polymers that swell at neutral pHs. Kim et al. [54] reported that ethylcellulose coatings are able to retard the gastric and intestinal drug release, which is then gradually released in the colon. Drug release was impeded by the layer of ethylcellulose, regardless the acidity of the medium. In addition, Sadeghi et al. [55] reported that drug release rate from ethylcellulose matrices is greatly affected by the amount of polymer used in the matrices formed, decreasing when the polymer concentration is increased.

On the contrary, β-carotene was not detected after the gastric phase of digested zein NPs. This result reveals the stability of these NPs in acidic pH and in the presence of pepsin. Zein is known by its rather low digestibility [56], being the resistance of zein to pepsin activity previously reported [57]. Also, zein NPs showed increased bioaccessibility values of β-carotene (37.0%) in the intestinal phase. Despite the resistance of zein to gastric conditions, this protein was reported to be hydrolysed by pancreatin under simulated intestinal conditions following the INFOGEST protocol [56]. These authors found that α-zein proteins degradates, according to a low number of peptides been identified at the end of the intestinal phase, which were not even detected by SDS-PAGE analysis.

Regarding the solvent extraction required, after digestion, to quantify β-carotene, the low solubility of zein in aqueous solution has limited the procedure compared to ethylcellulose nanoparticles. To improve the β-carotene extraction efficiency, two pre-treatments of the nanoparticles, previously reported [58], were tested. The pre-treatments performed were the incubation of zein loaded NPs with DMSO or with a bacterial protease (Alcalase). They did not improve the yields of β-carotene extraction (data not shown). The hydrolysis of zein using different bacterial proteases has been reported [59], suggesting the need of more harsh conditions, including higher temperatures, times of incubation or even mechanical disruption. However, this carotenoid is a thermolabil compound and extreme conditions might lead to its chemical denaturation. For this reason, the extraction efficiency was considered for calculating the bioaccessibility of the β-carotene.

The higher values obtained for zein NPs can be explained by the improved protection of β-carotene and lower release in the gastric phase and efficient incorporation within mixed micelles in the intestinal phase. Results are in agreement with micellarisation efficiency presented by Cheng et al. [40] for lutein loaded zein nanoparticles. Also, Mahalakshmi et al. [32], using β-carotene loaded zein nanoparticles showed bioaccessibility values of 31.4%.

According to the results obtained, zein nanoparticles showed to be the best option for the delivery of β-carotene when compared to ethylcellulose NPs. β-carotene loaded zein nanoparticles present higher bioaccessibility than the ones obtained for β-carotene rich foods [60], and therefore can be considered an effective way of delivering β-carotene in the intestinal phase. In 2007, Huo et al. [61] tested the effect of the addition of oil in meal samples (i.e., western-type salad) to improve the bioaccessibility of β-carotene, reaching a maximum of 18% of bioaccessibility when C:18:3 was used as fatty acid. 

## 3. Materials and Methods

### 3.1. Materials

Ethylcellulose with a viscosity ranged between 40 to 52 cP, molecular weight of 160.0 g/mol and an ethoxyl content of 48 to 49.5% was obtained from Ashland (Wilmington, DE, USA). Zein from maize seeds with 35% of α-zein (with 2 prominent bands of 22 and 24 kDa) and β-carotene (Type II, synthetic, ≥95% (HPLC), crystalline) were purchased from Sigma Aldrich (Saint Louis, MO, USA). β-carotene was kept at −22 °C and in the darkness until use. Ethanol absolute was obtained from Honeywell Riedel-de-Haën (Muskegon, MI, USA). Pure water was obtained by a Milli-Q system (Merck, S.A., Algés, Portugal). Pepsin from porcine gastric mucosa (EC Number 232-629-3), hemoglobin from bovine blood, sodium chloride, calcium chloride, pancreatin from porcine pancreas (EC Number 232-468-9), TAME (Nα-p-Tosyl-L-arginine methyl ester hydrochloride), Trizma base and bile bovine were purchased from Sigma-Aldrich (Saint Louis, MO, USA). The concentration of bile acids in bile were measured with a commercial assay kit also obtained from Sigma-Aldrich (Saint Louis, MO, USA). Trichloroacetic acid was obtained from Sigma-Aldrich (St. Louis, Missouri, EUA). Hydrochloric acid purchased from ThermoFisher Scientific (Waltham, MA, USA).

### 3.2. Production of Nanoparticles (NPs)

The production of the NPs followed the methodology described by Fessi et al. [4] based on the interfacial polymer deposition following solvent displacement. According with the biopolymer used, different approaches were followed based on preliminary studies (results not shown). For the production of ethylcellulose NPs, ethylcellulose (0.1, 0.2 or 0.4 g) was dissolved in 100 mL ethanol for at least 4 h at room temperature (≈22 °C) under stirring (300 rpm). Afterwards, deionized water was added to organic phase under continuous magnetic stirring (240 rpm) by means of a pipette aiming a final antisolvent concentration of 60, 70 and 80% (*v/v*). The solution was kept stirring for 15 min at 240 rpm. To produce β-carotene loaded ethylcellulose NPs, the organic phase was prepared by dissolving 0.1 g of ethylcellulose and 4 × 10^−4^ g of β-carotene in 100 mL ethanol at room temperature and then the same methodology described for the production of unloaded ethylcellulose NPs was followed. After the production of the nanoparticles, the solvent was evaporated using a rotary evaporator (IKA^®^-Werke GmbH & CO. KG, Staufen, Germany) at 60 °C, under reduced pressure of at least 30 mbar until ethanol was removed (step only performed for the optimized conditions). For the production of zein NPs, zein (0.4, 0.6 or 0.8 g) was dissolved in 100 mL of ethanol solution (75%, *v/v*) at least for 4 h at room temperature (≈22 °C) under stirring (300 rpm). The zein solution was then added dropwise to water using a syringe pump (New Era Pump Systems Inc., Farmingdale, NY, USA) at a constant stirring rate of 235 rpm aiming a final antisolvent concentration of of 80, 85 and 90% (*v/v*). The solution was then kept for 15 min at 235 rpm. To produce β-carotene loaded zein NPs two different solutions were made for the preparation of the organic phase: (i) 0.5 g of zein was dissolved in 100 mL of 75% aqueous ethanol solution and (ii) 2.5 × 10^−5^ g of β-carotene was dissolved in 50 mL of 100% ethanol (0.005% β-carotene concentrated solution). The two solutions were mixed by adding 10 mL of the β-carotene solution to 40 mL of zein solution under constant stirring at 200 rpm thus making the final organic phase with a final concentration of ethanol of 75% (*v/v*). Then, the same methodology described before for the production of unloaded zein nanoparticles was followed. After the production of the nanoparticles, the solvent was evaporated using a rotary evaporator as explained above (step only performed for the optimized conditions). To obtain the maximum β-carotene loaded NPs increasing β-carotene loaded concentrations were tested. The concentrations were based in the maximum carotene solubility in ethanol reported of 30 μg/mL [62], and on the ability of producing NPs with similar particle size and PDI that the unloaded NPs. The concentrations of β-carotene tested ranged from 400 µg/mL to 0.8 µg/mL for ethylcellulose NPs and from 400 µg/mL to 5 µg/mL for zein NPs.

### 3.3. Nanoparticle Characterisation

#### 3.3.1. Particle Size, Polydispersity Index and Surface Potential

The particles mean size by intensity, polydispersity index (PDI) and zeta potential were determined using dynamic light scattering (Nanopartica SZ-100, Horiba, Kyoto, Japan). For size and PDI determination were used a disposable cuvette with four openings and for zeta potential was used a carbon electrode cell. All measurements were performed at temperature of 25 °C, actively maintained within 0.1 °C in the sample chamber. The samples were irradiated with diode pumped frequency doubled laser (532 nm, 10 mW) and the intensity fluctuations of the scattered light were detected at angle of 90°. The particle refractive index was used 1.59 and 1.45 for ethylcellulose and zein, respectively, and dispersion medium used was water with a refractive index of 1.33. The software (Horiba SZ-100Z Type) determined the size mean the according with diffusion coefficient using the Stokes-Einstein equation and the potential zeta values were calculated by Smoluchowsi’s model. The measurements were performed immediately after the production process without dilute the samples. No sedimentation was observed during the measurement. For each sample at least three measurements were performed.

#### 3.3.2. Encapsulation Efficiency

Encapsulation efficiency was determined as presented elsewhere [63] with some modifications. The methodology was modified by increasing the time and centrifugal force used in order to guarantee that all the NPs sediment and were not in suspension. Freshly produced nanoparticles were submitted to ultracentrifugation for the separation of nanoparticles from free non-encapsulated β-carotene using an Ultracentrifuge (OPTIMA XE-100, Beckman Coulter Life Sciences, Indianapolis, IN, USA). The nanoparticle solution was centrifuged at 257,300× *g*, for 1 h. The β-carotene dispersed in the supernatant was analysed spectrophotometrically in a Microtiter plate reader (Synergy H1, BioTek Instruments, Winooski, VT, USA) at 453 nm in triplicate using a blank solution of pure ethanol. The encapsulation efficiency for both ethylcellulose and zein NPs was calculated by the following equation:

(1) $$\mathrm{EE}\;(\%)\;=\;\frac{{\mathrm{BC}}_{\mathrm{INITIAL}}\;-\;{\mathrm{BC}}_{\mathrm{FREE}}}{{\mathrm{BC}}_{\mathrm{INITIAL}}}\times 100$$

Being EE (%) the percentage of encapsulation efficiency, ${\mathrm{BC}}_{\mathrm{INITIAL}}$ the initial concentration of β-carotene in the particle suspension, and ${\mathrm{BC}}_{\mathrm{FREE}}$ the concentration of β-carotene in suspension after centrifugation.

#### 3.3.3. Transmission Electron Microscopy

Morphology was evaluated through a transmission electron microscope (JEM-2100, JEOL Ltd., Tokyo, Japan) operating at 200 kV accelerating voltage. TEM micrographs were analysed using the public domain software ImageJ. Two images of each sample were analysed, being measured at least ten particles by each image. A drop of the sample solution (≈10 μL) was placed on a grid (ultra-thin carbon film on Lacey carbon support film, 400 mesh, Copper, Ted Pella Inc., Redding, CA, USA). Then, contrast solution UranyLess EM Stain (Electron Microscopy Sciences (EMS), Hatfield, PA, USA) was dropped on parafilm and the grid was placed on top of the drop so it could be stained. It was left to dry for approximately 5 h at room temperature.

#### 3.3.4. Fourier Transform Infrared (FTIR) Spectroscopy

Measurements were made using a FTIR VERTEX 80/80v spectrometer (Bruker Corporation, Billerica, MA, USA) in Attenuated Total Reflectance mode (ATR) with a platinum accessory and a diamond crystal with a refractive index of 2.4, in the wavelength range: 4000–400 ${\mathrm{cm}}^{-1}$, using 32 scans at a resolution of 4 ${\mathrm{cm}}^{-1}$. Before analysis, an open bean background spectrum was recorded as a blank and used for baseline correction. All data is presented in transmittance percentage after a normalisation to the maximum transmittance value. For each sample one measurement was performed.

#### 3.3.5. X-ray Diffraction

An X-ray diffraction system (Malvern Panalytical Ltd., Malvern, UK) was used to evaluate the crystallographic structure. PANanalytical X’Pert HighScore Plus was the software used to gather data and analyse peak diffractions. Background noise was also measured. The powder sample was added to the glass slide through an adhesive glue and put on the sample holder for detection. The XRD diffractograms were acquired at room temperature, angular scans from 5° to 50° (2θ) were performed with a Cu source, X-ray tube (λ = 1.54056 Å) at 45 kV and 40 mÅ. The fine calibration offset for 2θ = −0.0372°. For each sample one measurement was performed.

### 3.4. Bioaccessibility In Vitro Gastrointestinal Digestion

#### 3.4.1. In Vitro Digestion

The in vitro digestion protocol followed in this work was established by the COST INFOGEST network [51] where the sample is subjected to three sequential phases: oral, gastric and intestinal.

Initially, the enzymes, bile and stock solutions were prepared. The digestion involves the action of enzymes, such as amylase, pepsin, lipase, trypsin and chymotrypsin. The pepsin activity and trypsin activity (in pancreatin) were determined to calculate the amount (mg/mL) needed of each enzyme in the in vitro digestion. Amylase was not used since there was no starch present in the digested sample. The addition of gastric lipase was omitted due to the limited access of the commercially available enzyme. Pepsin activity assay is based on the spectrophotometric stop reaction method. One unit will produce a ΔAbs_280_ of 0.001 per minute measured at TCA-soluble products (pH 2 and 37 °C) [51]. Two different solutions were prepared previously to the assay: substrate and enzyme solutions.

To measure the trypsin activity in pancreatin the kinetic spectrophotometric rate determination method was used. One unit corresponds to the hydrolysis of 1 μmol of TAME per minute (pH 8.1 and 25 °C) [51]. Bile acids concentration was measured following the supplier’s protocol which provides a fluorometric method to measure the total bile acids.

The stock electrolytic solutions were prepared according to the INFOGEST protocol [51]. Simulated salivary fluid (SSF), simulated gastric fluid (SGF) and simulated intestinal fluid (SIF) were prepared 1.25 times concentrated, considering the later dilution (4:1) with enzymes and CaCl_2_(H_2_O)_2_ added just before the assay to avoid precipitation.

Afterwards, three different samples were submitted to the digestion procedure: ethylcellulose NPs loaded with β-carotene, zein NPs loaded with β-carotene and a blank using water. All samples containing β-carotene were prepared in order to achieve an initial concentration of β-carotene of 20 μg/mL. All samples were digested in triplicate. In this study, the digestion was evaluated at two time points: after the gastric phase and after the intestinal phase. In the oral phase the sample was diluted 1:1 (*v/v*) with simulated salivary fluid, calcium chloride and water. Briefly, to 5 mL of each sample, 4 mL of SSF, 25 μL CaCl_2_(H_2_O)_2_ 0.3 M and 0.975 mL of water were added. The tubes were incubated in an orbital incubator (Fisher Scientific) for 2 min at 37 °C and 150 rpm. For the gastric phase, a pepsin solution 13.84 mg/mL (2000 U/mL) in water was prepared based on the activity previously determined. The 10 mL of oral phase obtained in the former phase were diluted 1:1 (*v/v*) with 8 mL of SGF, 1 mL of pepsin solution, 5 μL CaCl_2_(H_2_O)_2_ 0.3 M, 300 μL HCl 1 M and 695 μL of water. The pH was adjusted to 3.0 using HCl. The samples were for 2 h at 37 °C and 150 rpm. For the intestinal phase, bile solution 60 mg/mL and pancreatin 148.15 mg/mL (100 U/mL) were prepared in SIF. The 20 mL of gastric phase were diluted 1:1 (*v/v*) with 6.57 mL SIF, 5 mL of pancreatin solution, 4.43 mL of bile, 40 μL CaCl_2_(H_2_O)_2_ 0.3 M, 140 μL HCl 1 M and 3.82 mL of water. The pH was adjusted to 7.0. The samples were incubated for 2 h at 37 °C and 150 rpm.

#### 3.4.2. β-Carotene Extraction

For the β-carotene quantification, digested samples were centrifuged for 20 min at 4 °C and 3 100× *g* to collect the supernatant (assumed as micellar phase), followed by solvent extraction using the methodology reported by Wright et al. [52]. After centrifugation, 500 μL of supernatant was transferred to a tube to begin the extraction. The solvents were added: 0.5, 3.0 and 1.0 mL of ethanol, acetone and distilled water, respectively, vortexing for 10 s between each addition. Then, 2 mL of hexane was added, the tubes were mixed by inversion and rested until a phase separation was observed. Then, the top organic layer was removed, and three sequential extractions with 1 mL of hexane were performed. The organic phases were pooled together (~5 mL of hexane) and evaporated (Modular Centrifugal Evaporator, Fisher Scientific, Lda, Porto Salvo, Portugal) at 40 °C under vacuum until a volume of approximately 500 μL was left. All volumes were set to 500 μL adding the volume of fresh hexane needed and transferred to vials for posterior analysis. Two pretreatments were tested before extraction of β-carotene from zein NPs. The pretreatments were intended to disrupt the NPs promoting the release of the encapsulated β-carotene. They consisted in the addition to 0.5 mL of NPs suspension of: (1) 1 mL of DMSO followed by 1 min vortex agitation (repeated 3 times, with ice cooling within repeats), or (2) 1 mL of Alcalase 2.4 L (Novozyme Nordisk, Bagsvaerd, Denmark) in PBS pH 7.4 (0.024 U/mL) and incubation for 10 min at 45 °C. Then β-carotene was then extracted using the same procedure described above.

In order to control the extraction efficiency, β-carotene was extracted from non-digested β-carotene loaded zein nanoparticles. The initial dispersion was diluted into three different β-carotene concentrations (10.0, 5.0 and 2.5 μg/mL) and the extraction was performed. Extraction efficiency was calculated using Equation (2):

(2) $$\mathrm{Extraction}\;\mathrm{efficiency}\;(\%)\;=\;\frac{C_{E}}{C_{0}}\;\times \;100$$

C_E_ being the concentration of β-carotene extracted and C_0_ the initial β-carotene concentration.

#### 3.4.3. β-Carotene Quantification and Bioaccessibility

The β-carotene was quantified by high liquid performance chromatography (HPLC) using an Agilent 1260 Infinity Quaternary LC (Agilent Technologies, Santa Clara, CA, USA) equipped with a Kinetex 2.6 µm XB-C18 column (150 × 4.6 mm, Phenomenex, Torrance, CA, USA). The mobile phase was composed of methanol and acetonitrile (90:10) under a flow rate of 1.8 mL/min, and the injection volume was 50 µL. The β-carotene was eluted and monitored with a Diode Array Detector (DAD, Agilent Technologies, Santa Clara, CA, USA) at 450 nm, being the retention time of 4.2 min and the quantification limit of 0.156 μg/mL. Then, the area of the β-carotene peak in the samples was compared with the areas of a calibration curve constructed using a series of β-carotene standard (Sigma-Aldrich, St Louis, MO, USA) solutions prepared in hexane at concentrations ranging from 0.15 to 25 µg/mL. Therefore, the concentration of β-carotene was obtained after interpolation of the area of the samples in the calibration curve, being the mass of β-carotene calculated given the digestion volume (20 mL or 40 mL for gastric or intestinal phase, respectively). The bioaccessibility of encapsulated β-carotene after digestion was calculated using the following equation:

(3) $$\mathrm{Bioaccessibility}\;(\%)\;=\;\frac{m_{\mathrm{micelle}}}{m_{\mathrm{initial}}}\;\times \;100$$

Being $m_{\mathrm{micelle}}$ the mass of β-carotene in the micelle fraction after digestion and $m_{\mathrm{initial}}$ the initial mass of β-carotene.

### 3.5. Statistical Analysis

Two sets of experiments were performed for the production of the NPs. For ethylcellulose NPs the independent variables were the ethylcellulose and antisolvent concentrations, being used two levels and one central point (Table S1). In the case of zein NPs the zein concentration, antisolvent concentration and the flow rate were the independent variables, being used two levels and one central point (Table S2). The data obtained from the experimental design were subjected to a statistical analysis using Statistica software (release 7, edition 2004, Statsoft, Tulsa, OK, USA). Pareto charts were drawn to express the statistical significance of each factor and the interactions between factors visually.

## 4. Conclusions

β-carotene loaded in zein or ethylcellulose nanoparticles (size below 100 nm) were successfully produced using the solvent-displacement method. The nanoparticles presented spherical morphology and narrow size distributions, maintained after β-carotene encapsulation, reaching an encapsulation efficiency of 93% and 86% for zein and ethylcellulose nanoparticles, respectively, and similar particle size than the unloaded NPs. FTIR and XRD analysis showed that the main structure of the materials used are maintained, and that β-carotene is present in a more amorphous state when encapsulated. In vitro digestion study showed that both nanosystems are strongly insoluble in the digestion medium, hampering the action of the enzymes and salts to release the β-carotene from the nanoparticles. Both nanoparticles showed to protect the β-carotene in the gastric phase but only zein nanoparticles result in a good bioaccessibility values in the intestinal phase. Further in vitro studies should be performed to assess the possible NPs cytotoxicity and evaluate the intestinal absorption of β-carotene after the digestion, allowing the estimation of the effective bioavailability obtained through the β-carotene encapsulation.

## Figures and Tables

**Table 1:** Average size, PDI and zeta potential for unloaded and loaded ethylcellulose and zein nanoparticles (NPs).

Sample	Average Size (nm)	PDI	Zeta Potential (mV)
Ethylcellulose NPs	69 ± 2ab	0.18 ± 0.04a	−61 ± 2b
Loaded Ethylcellulose NPs *	60 ± 9a	0.27 ± 0.02b	−93 ± 3a
Zein NPs	82 ± 7bc	0.34 ± 0.05b	64 ± 2c
Loaded Zein NPs *	83 ± 8c	0.29 ± 0.06b	70.5 ± 0.7d

Values reported are the mean ± standard deviation (sd). Different letters (a–d) in the same column indicate a statistically significant difference (*p* < 0.05). * Ethylcellulose NPs were loaded with 4 µg/mL and zein NPs were loaded with 10 µg/mL.

**Table 2:** Bioaccessibility of β-carotene, after each digestion phase, for loaded ethylcellulose and zein nanoparticles (NPs).

Sample	Bioaccessibility (%)	
	Gastric Phase	Intestinal Phase
Loaded ethylcellulose NPs	2.7 ± 0.1	8.3 ± 0.1a
Loaded zein NPs	- *	37 ± 1b

Values reported are the mean ± standard deviation (sd) of three sample replicates. Different letters (a–d) in the same column indicate a statistically significant difference (*p* < 0.05). * β-carotene was detected in one sample out of the three digestion replicates, being the calculated concentration below the quantification limit.

## Supplementary Materials

The following are available online, Table S1. Average size and polydispersity (PDI) of ethylcellulose nanoparticles, Table S2. Average size and polydispersity (PDI) of zein nanoparticles.
