# Supplementary material for: Bio-Based Nanoparticles as a Carrier of β-Carotene: Production, Characterisation and In Vitro Gastrointestinal Digestion
Source: Molecules. 2020 Sep 30;25(19):4497. doi: 10.3390/molecules25194497 (PMC7582681; doi:10.3390/molecules25194497)
Supplement: Supplementary file 1 [file molecules-25-04497-s001.pdf]

Supplementary information

Table S1. Average size and polydispersity (PDI) of ethylcellulose nanoparticles

Ethylcellulose Concentration (%)	Antisolvent Concentration (%)	Average Size (nm)	PDI
0.1	60	163.6	0.20
0.1	80	99.83	0.29
0.4	60	258.3	0.24
0.4	80	187.5	0.25
0.2	70	166.3	0.15
0.2	70	193.8	0.14
0.2	70	188.2	0.17

Table S2. Average size and polydispersity (PDI) of zein nanoparticles

Zein Concentration (%)	Antisolvent Concentration (%)	Flow Rate (mL/min)	Average Size (nm)	PDI
0.4	80	0.3	131.0	0.21
0.4	80	0.7	136.7	0.20
0.4	90	0.3	91.0	0.23
0.4	90	0.7	95.5	0.29
0.8	80	0.3	156.1	0.18
0.8	80	0.7	162.3	0.21
0.8	90	0.3	104.5	0.24
0.8	90	0.7	104.1	0.29
0.6	85	0.5	142.9	0.22
0.6	85	0.5	116.7	0.22
0.6	85	0.5	125.5	0.23
